# Histopathology reporting of temporal artery biopsy specimens for giant cell arteritis: results of a modified Delphi study

**DOI:** 10.1136/jcp-2023-208810

**Published:** 2023-06-15

**Authors:** Dilek Taze, Arundhati Chakrabarty, Ranjana Venkateswaran, Collette Hartley, Charlotte Harden, Ann Wendy Morgan, Sarah Louise Mackie, Kathryn Jane Griffin

**Affiliations:** 1 Department of Histopathology, Leeds Teaching Hospitals NHS Trust, Leeds, UK; 2 Leeds Institute of Cardiovascular & Metabolic Medicine, University of Leeds, Leeds, UK; 3 Leeds Institute of Rheumatic and Musculoskeletal Medicine, University of Leeds, Leeds, UK; 4 Department of Rheumatology, Leeds Teaching Hospitals NHS Trust, Leeds, UK

**Keywords:** VASCULITIS, DIAGNOSIS, INFLAMMATION, NEUROPATHOLOGY, Pathology, Surgical

## Abstract

The temporal artery biopsy (TAB) is regarded as the gold-standard test in the diagnosis of giant cell arteritis (GCA). There is a lack of agreement among experienced pathologists regarding the diagnostic features and classification of inflammation observed in TAB sections in the diagnosis of GCA.

**Aims:**

The aim of this research study was to establish consensus on the key parameters which should be included in a standardised reporting proforma for TAB specimens. We specifically investigated factors pertaining to clinical information, specimen handling and microscopic pathological features.

**Methods:**

A modified Delphi process, comprising three survey rounds and three virtual consensus group meetings, was undertaken by 13 UK-based pathology or ophthalmology consultants, with a 100% response rate across the three rounds. Initial statements were formulated after a literature review and participants were asked to rate their agreement using a nine-point Likert scale. Consensus was defined a priori as an agreement of ≥70% and individual feedback was provided after each round, together with data on the distribution of group responses.

**Results:**

Overall, 67 statements reached consensus and 17 statements did not. The participants agreed on the core microscopic features to be included in a pathology report and felt that a proforma would facilitate consistent reporting practices.

**Conclusions:**

Our work revealed uncertainty surrounding the correlation between clinical parameters (eg, laboratory markers of inflammation and steroid therapy duration) and microscopic findings, and we propose areas for future research.

**WHAT IS ALREADY KNOWN ON THIS TOPIC**

- The temporal artery biopsy is the gold standard for diagnosis of giant cell arteritis (GCA) yet there is lack of agreement about classification and histopathologists are unsure as to which features should be reported.

**WHAT THIS STUDY ADDS**

- This study uses a Delphi method to resolve important areas of disconcordance in pathology practice for the reporting of GCA.

**HOW THIS STUDY MIGHT AFFECT RESEARCH, PRACTICE OR POLICY**

- This work could be used as the basis for a standardised reporting template and has highlighted areas where more research is needed.

## Introduction

Reportedly first described by Hutchinson in 1890,^1^ giant cell arteritis (GCA) is a serious form of vasculitis with a predilection for older individuals. GCA typically affects the temporal arteries, hence the alternative name of ‘temporal arteritis’ and can result in irreversible ischaemic complications including blindness.

Patients with suspected GCA are managed with high dose glucocorticoids and remain on this precautionary treatment until a diagnosis is confirmed.^2^ Prompt and definitive diagnosis is challenging due to the non-specific and varied clinical presentation and the lack of a robust diagnostic biomarker.^2^

The temporal artery biopsy (TAB) is regarded as the gold-standard test in GCA diagnosis and forms part of the core diagnostic criteria in all UK, European and American classification guidelines.^2–4^ Under current National Health Service (NHS) England prescribing guidance, a TAB result is a component of the eligibility criteria for the biologic therapy tocilizumab.^5^ Despite the recognised importance of this test, a recently published UK-wide audit of experienced pathologists found a lack of agreement on the diagnostic features and classification of inflammation observed in TAB sections for the diagnosis of GCA.^6^ Of the nine micrographs circulated for assessment, only one reached complete agreement in terms of ‘bottom-line’ histopathological diagnosis.

To further determine the extent of variation and uncertainty in the reporting of TABs for the diagnosis of GCA, our research group Atlas of Histopathology Education for Advancing Diagnostics in GCA conducted an online survey in June 2021 of all consultant members of the Royal College of Pathologists (RCPath).^7^ We received 116 responses with 60% having ≥15 years of consultant clinical practice. Forty-two per cent of respondents expressed some level of uncertainty regarding the histological reporting criteria for diagnosing GCA.^7^ A consistent theme in the comments provided by participants was the lack of clear consensus as to what features constitute a GCA diagnosis. Nearly two-thirds of respondents were in support of the development of a standardised pathology TAB reporting template as part of an RCPath tissue pathway. The key survey results can be found in the supporting information (online supplemental figure 1s).

10.1136/jcp-2023-208810.supp1 Supplementary data

The implications of misdiagnosis in GCA patients include delay or prevention of alternative diagnosis and/or unnecessary exposure to glucocorticoids and their potential harmful side effects (false positive), or denied access to tocilizumab therapy, which requires histological or imaging diagnostic confirmation in the UK (false negative). Despite an increasing use of ultrasound (US) for non-invasive diagnosis,^2^ the TAB remains a critical investigation in GCA worldwide, and thus there is a clear need to address the issue of disconcordance between histopathologists in the assessment and reporting of TABs for the diagnosis of GCA.

The Delphi method is a well-established research methodology, which uses an expert panel for the purpose of generating consensus in instances where empirical evidence is limited or contradictory.^8^ Work from our group has shown that the Delphi methodology can be used in histopathology to reach consensus in a range of circumstances, including, but not limited to, determining the pertinent information to be included in pathology reports.^9^ The aim of this Delphi study was to reach consensus on the following key parameters for the reporting of TAB specimens for the diagnosis of GCA: (1) clinical information provided on the request form, (2) specimen handling of TABs and (3) key microscopic features to be included in the pathology report.

## Materials and methods

A modified Delphi study, comprising three survey rounds and three virtual consensus group meetings, was undertaken between December 2021 and April 2022.

### Expert panel selection

Histopathologists who completed the consultant RCPath survey and expressed interest in being part of an expert consensus group were contacted for invitation to participate in the modified Delphi study. While there is intrinsic bias in recruiting individuals who express interest in the area of study and are therefore more likely to be affected by the outcomes, the Delphi method is a time-intensive process and requires motivated participants who will commit to the duration of the study.^10^ Individuals known to the authors from other clinical specialities with expertise and interest in GCA were also contacted. Only participants working in the UK were eligible for inclusion and in total there were 13 consultant participants comprising 11 histopathologists and 2 ophthalmologists, with a mean number of years of consultant experience of 16.9 years (range: 1 month to 29 years). This consensus group represents the diversity of the user group who report and/or interpret the TAB pathology reports for clinical purposes and will be referred to as the ‘expert panel/participants’ for the purpose of this Delphi. These individuals are listed in the Acknowledgements section, and we are grateful for their valued contribution to our work.

### Statement formulation process

**Figure 1:**
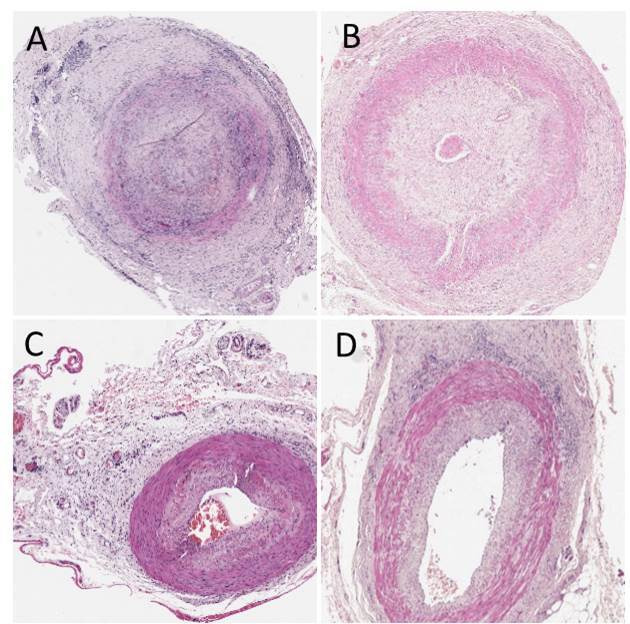
Example whole slide images used in the Delphi study.

The initial 80 statements were formulated after a comprehensive literature review and discussion between the primary authors including three histopathologists and two rheumatologists. Key papers relating to parameters of interest were collated and sent to the study participants for review before starting the Delphi process to standardise knowledge and maximise the efficiency of discussions.^11–16^ Figure 1 provides example whole slide images (WSI) hosted by the Leeds Virtual Pathology website^17^ (refer to online supplemental content).

### The Delphi process

Each survey cycle lasted for 3 weeks and was administered via the Leeds Online Surveys platform (see online supplemental content).

The participants were required to rate their level of agreement with each statement on a 9-point Likert scale (1=strongly disagree and 9=strongly agree). An ‘unable to score’ option and a free-text response box for comments was also provided for each statement (see online supplemental figure 2s). Consensus was defined a priori and was considered to have been reached if ≥70% of the expert participants were in agreement.

Individual feedback was provided to the participants showing their own rating to any statement which did not reach consensus, as well as the distribution of the group responses overall, and the anonymised free-text responses (see supporting information online supplemental figure 3s). The purpose of this feedback was to give the participants an opportunity to reconsider their previous ratings in the context of the distribution of group responses and is a recognised key element of the Delphi process.^18^

Statements which did not reach consensus were discussed at the end of the round period in an online consensus group meeting, chaired by the study co-ordinator. Rheumatology expert clinical opinion was provided by SLM and AWM during the focus group discussions. The participants were given the opportunity to reword/modify statements, add a new statement, ‘discard’ the statement (ie, consider it redundant) or add it to the research agenda. These statements were then circulated in the subsequent round. This methodology was repeated for survey rounds 2 and 3.

## Results

**Figure 2:**
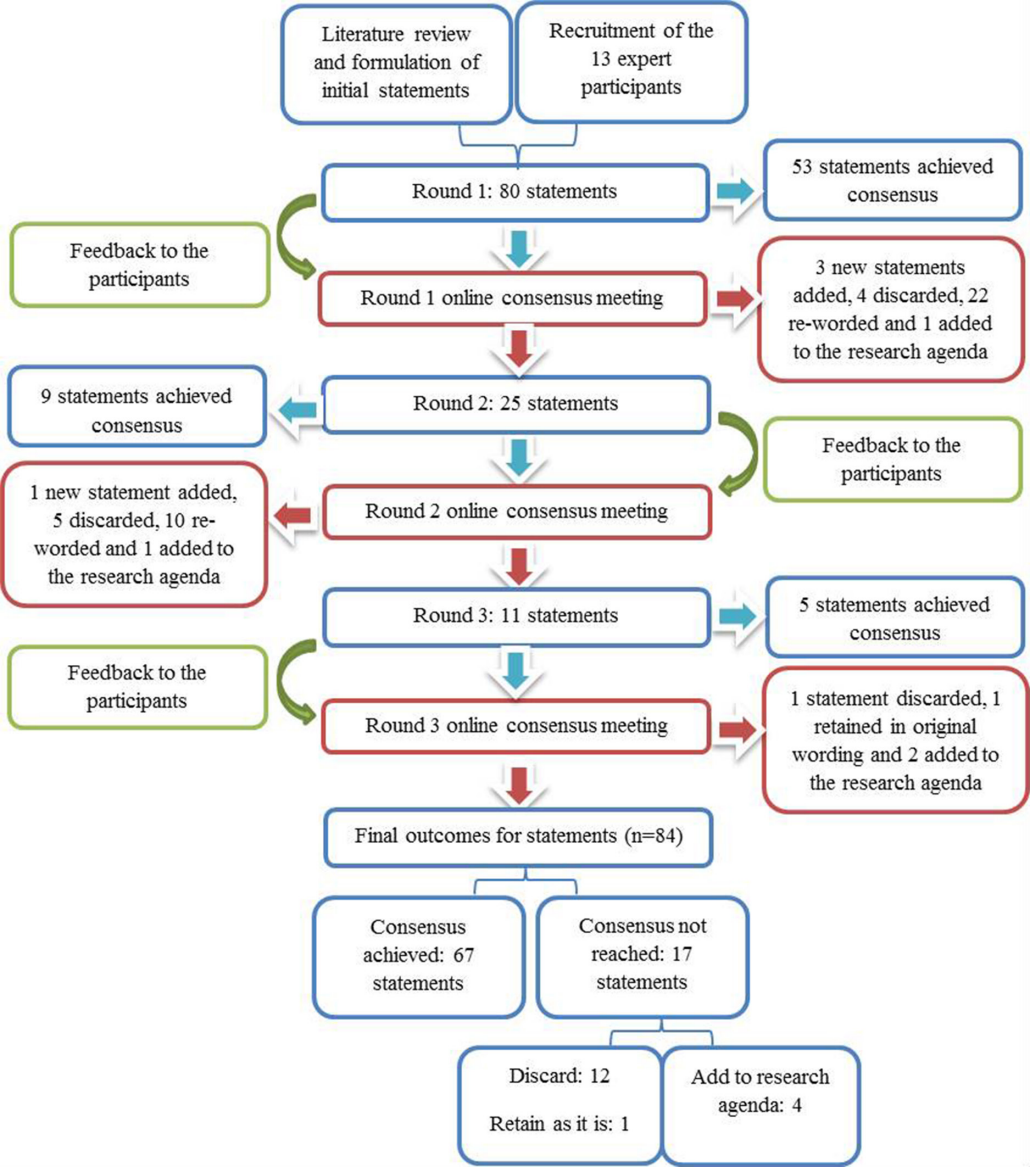
Flow chart illustrating the modified Delphi process.

The first survey round comprised 80 statements across the following categories or themes: (1) clinical information, (2) specimen handling and tissue pathways, (3) microscopic assessment and reporting and (4) WSI-based statements. Fifty-two statements reached positive consensus (≥70% agreement), 1 statement reached negative consensus (≥70% disagreement) and was subsequently removed, and 27 statements did not reach consensus. Figure 2 illustrates the Delphi process and summarises the overall outcomes for each round.

**Figure 3:**
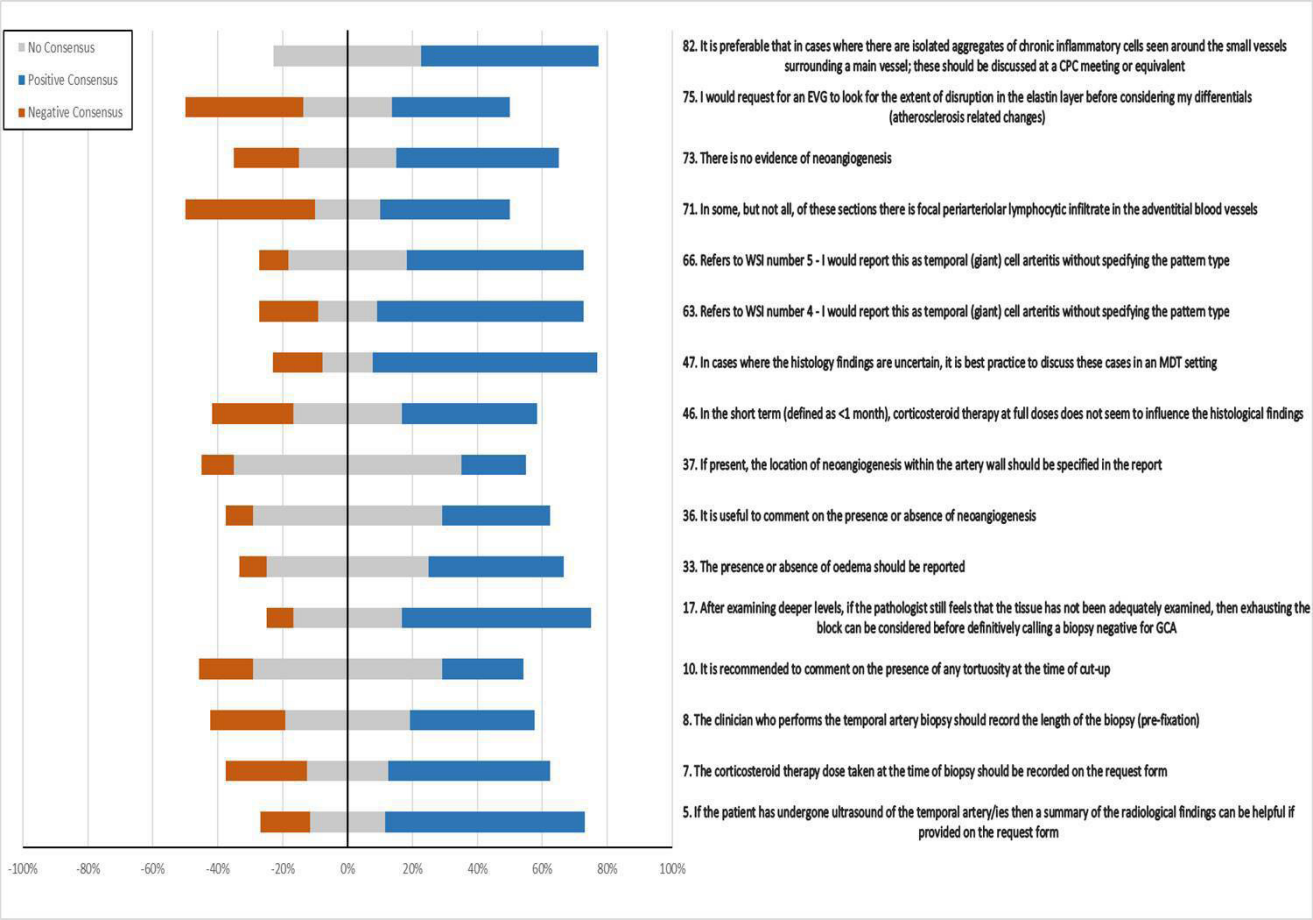
Level of agreement with the statements that did not reach consensus. GCA, giant cell arteritis; WSI, whole slide images; CPC, clinicopathological correlation.

In total, 84 statements were rated±discussed during the three survey rounds. The response rate was 100% (13/13 participants) for all three rounds. The list of 67 statements reaching consensus is shown in online supplemental table 1s as supporting information. There were 17 statements that did not achieve consensus, and these are listed in table 1 with their consensus meeting outcomes and figure 3 illustrates the level of agreement to these statements. These are discussed below (number in parentheses relates to the Delphi statement number):

The expert panel reached consensus on using a classification system that recognises the different patterns of GCA as important for improving standardisation of the reporting of GCA, as described by Hernández-Rodríguez *et al.*(39) Consensus was reached on the reporting of the following microscopic features for all TABs for GCA diagnosis: the location and extent of inflammatory cells and granulomatous infiltration in the artery wall (23, 30), and the presence or absence of intimal hyperplasia (31), fibrosis (32) and luminal occlusion (34). In addition, the panellists agreed that it can be useful to comment on whether the tunica media is intact or disrupted (24).

**Table 1:** List of statements that did not achieve consensus and their outcomes

No	Statement	Agreement	Outcome
5	If the patient has undergone ultrasound of the temporal artery/ies then a summary of the radiological findings can be helpful if provided on the request form	62%	Retain
7	The corticosteroid therapy dose taken at the time of biopsy should be recorded on the request form	50%	Discard
8	The clinician who performs the temporal artery biopsy should record the length of the biopsy (prefixation)	38%	Discard
10	It is recommended to comment on the presence of any tortuosity at the time of cut-up	25%	Discard
17	After examining deeper levels, if the pathologist still feels that the tissue has not been adequately examined, then exhausting the block can be considered before definitively calling a biopsy negative for GCA	58%	Discard
33	The presence or absence of oedema should be reported	42%	Discard
36	It is useful to comment on the presence or absence of neoangiogenesis	33%	Add to research agenda
37	If present, the location of neoangiogenesis within the artery wall should be specified in the report	20%	Discard
45	I find the terms ‘healed’ or ‘treated’ arteritis are unhelpful for clinicians	38%	Discard -combined with statement 44
46	In the short term (defined as<1 month), corticosteroid therapy at full doses does not seem to influence the histological findings	42%	Add to research agenda
47	In cases where the histology findings are uncertain, it is best practice to discuss these cases in an MDT setting	69%	Discard
63	Refers to WSI number 4—I would report this as temporal (giant) cell arteritis without specifying the pattern type	64%	Add to research agenda
66	Refers to WSI number 5—I would report this as temporal (giant) cell arteritis without specifying the pattern type	55%	Discard
71	In some, but not all, of these sections there is focal periarteriolar lymphocytic infiltrate in the adventitial blood vessels	40%	Discard
73	There is no evidence of neoangiogenesis	50%	Discard
75	I would request for an EVG to look for the extent of disruption in the elastin layer before considering my differentials (atherosclerosis related changes)	36%	Add as research agenda
Additional statements added by the expert participants			
82	It is preferable that in cases where there are isolated aggregates of chronic inflammatory cells seen around the small vessels surrounding a main vessel; these should be discussed at a CPC meeting or equivalent	55%	Discard

CPC, Clinicopathological correlation; EVG, Elastic Van Gieson; MDT, Multidisciplinary team.

Areas for further research were identified as follows: (1) whether there are any clinical associations to support the reporting of neoangiogenesis, (2) to what extent do the various patterns of GCA correlate with clinical parameters and (3) is there a direct, and measurable relationship between the duration of steroid therapy treatment and the degree of chronic inflammation observed on biopsy.

Further information on the key outcomes from each of the four themes is discussed, in detail, in online supplemental information section.

## Discussion

The TAB remains a critical tool in the clinical evaluation and therapeutic decision-making process for patients with suspected GCA. Despite this, recent literature suggests that there is a lack of agreement among experienced pathologists on the diagnostic features and classification of inflammation observed in TAB sections. We aimed to identify which clinical and histological parameters are of diagnostic value for the reporting of TABs for GCA diagnosis. By means of a three-round modified Delphi study, consensus was reached by an expert panel on the key items to be included in a standardised reporting protocol.

The participants reached consensus on the minimal clinical information that is advisable for the clinician to provide on the request form. A common theme from all the agreed clinical parameters—but especially for US—was the uncertainty regarding how they correlate with, or predict, the histology findings. Outcome Measures in Rheumatology group defined the halo sign (homogeneous, hypoechoic wall thickening) as the characteristic finding of GCA on US.^19^ The TABUL (Temporal Artery Biopsy vs Ultrasound in Diagnosis of GCA) study is a multicentre, prospective study which followed 381 patients with suspected GCA, who were commenced on steroid treatment, and underwent TAB as well as US of both the temporal and axillary arteries.^16^ TABUL demonstrated that although the halo sign on US is less specific than TAB (77% vs 81%), it had a similar sensitivity compared with TAB (93% vs 91%). Furthermore, a subanalysis of the TABUL study cohort showed that jaw claudication and visual symptoms were more frequent in patients with the halo sign.^20^ At present, the European League Against Rheumatism recommends US as the first-line study for suspected GCA, however; most of our expert participants were not aware of their centre adopting this practice, or if routine US was even accessible to their clinicians.^3^ Given the increasing role of US in the diagnosis of GCA and the difficulty in relying solely on TABs for a diagnosis in nuanced cases, the participants agreed that standardisation of the minimum information provided by the clinicians on the TAB request form would improve current audits of practice and would facilitate the collection of data for future research.

High-dose corticosteroid therapy is the mainstay treatment for GCA and is important for reducing the risk of ischaemic-related complications. The duration of corticosteroid therapy was deemed the single most important clinical parameter and some participants felt strongly that steroid treatment can rapidly influence histological features. A landmark paper was shared with the expert panel prior to the start of the study to ‘debunk’ the notion that steroid treatment for several days, or even weeks, will result in a false-negative biopsy. Maleszewski *et al* reported the results of a first-of-its-kind prospective study of second TABs that were randomly assigned to time-period cohorts over a 1-year period.^15^ These results showed that despite steroid therapy, the inflammation observed in GCA persisted and the longest interval between steroid initiation and the histological presence of GCA was 420 days. These findings were debated during the first consensus group meeting, and while the participants decided that more research is needed in this area, it was agreed that steroid therapy dose, and start date, should be included as part of the minimum information provided on the request form.

The RCPath cardiovascular tissue pathway recommends commenting on tortuosity when assessing TABs at macroscopy.^11^ The diagnostic relevance and evidence base of this was questioned by the participants as there is no known literature to support this practice in pathology, although studies have demonstrated the value of this practice in temporal artery US. Monti *et al* have defined the parameters to be assessed when undertaking temporal artery US, which include commenting on significant tortuosity in instances where US interpretation is challenging.^21^ The rationale for this is that significant tortuosity can impact the reliability of the US assessment.

There was support for using a classification system that recognises the different patterns of GCA as important for improving standardisation of the reporting of GCA. Discussions took place to explore the best way of capturing this information on a pathology report. Some individuals expressed a preference for a ‘free-text’ or descriptive report for elaborating on the pattern of GCA, especially in instances where different sections of the same biopsy may show overlap between these patterns, in keeping with the proposed mechanism of GCA disease progression. Other participants were in favour of a standardised reporting template, with a ‘free text’ option, to enable consistent reporting practices and facilitate the collection of relevant research data. The participants expressed that the ‘free text’ option would be particularly helpful in instances where there is no active inflammation, but atypical findings are seen which need to be communicated. The panel felt that the terms ‘healing/healed/resolving/treated’ arteritis should be avoided in such cases, as it was agreed that these terms lack clear definitions and can be misinterpreted. Nevertheless, the participants emphasised that there needs to be education and training among pathologists in recognising the different GCA patterns for a standardised reporting template to be fit for purpose.

The participants agreed on the core microscopic features which should be included in the reporting of all TABs for GCA diagnosis. One feature which sparked debate among the panellists was ‘neoangiogenesis’ and whether this needs to be captured as a non-core item in a standardised reporting proforma. Dinkin and Johnson describe the pathogenesis of GCA and outline how platelet-derived growth factor and vascular endothelial growth factor, released by giant cells can cause intimal hyperplasia and neoangiogenesis, ultimately leading to vascular occlusion and the ischaemic complications of GCA.^22^ Several studies have reported an association between intimal thickening and the development of neuro-ophthalmic ischaemic complications,^23–25^ with one large study suggesting an association between neoangiogenesis and irreversible visual loss.^23^ Conversely, a different, although older study, with specific staining for endothelial cells has suggested that a significant degree of neoangiogenesis offers protection against GCA-induced ischaemic complications.^26^ The expert panel agreed that although further evidence is needed to reach consensus on the clinical implications of neoangiogenesis, there was also support for listing this item as a non-core item in a standardised reporting proforma to facilitate the data collection process for future research endeavours. Our Delphi study has also highlighted the need to investigate if, and how, the histological patterns of GCA correlate with various clinical parameters and the requirement to establish and validate the histological features of age-related change and atherosclerosis.

## Limitations

Our method differs from a ‘classical’ Delphi study in that online consensus meetings were incorporated to allow for contentious topics to be discussed and debated, paving the way for new ideas to be generated. An online platform was also preferred to in-person meetings due to COVID-19 restrictions and potential travel disruptions. The benefit of virtual consensus group meetings was that geographically dispersed experts were able to come together more easily. Anonymity and the avoidance of dominant characters are crucial factors to be accounted for in the Delphi method.^27^ We sought to address these factors by providing individual feedback to each participant with all comments and suggestions anonymised. While it is not always possible to prevent dominant characters from exerting undue influence in a group meeting, a summary of the consensus meeting discussions was circulated to the participants at the end of each round, with opportunity for the participants to raise any thoughts or comments directly with the moderator.

One of the limitations of the Delphi method is the minimal published guidance on the formulation of statements.^28^ While Delphi statements are usually derived from the relevant literature and best practice guidelines, they are ultimately chosen and worded by the study co-ordinators. We sought to address this limitation by asking the participants for new statement suggestions and/or rewording of the existing statements at the end of each survey round. Our expert panel comprised UK-based consultants only, which limits the generalisability of our findings. While recruitment of an international panel may have increased the breadth of opinion, there are inherent differences between the UK’s NHS and other healthcare systems, and we sought to ensure that our findings could easily be translated to changes in guidelines and practice within the NHS. Likewise, while we recognise the intrinsic bias of recruiting panel members from those who ‘self-selected’ to complete our initial survey, the Delphi methodology is an involved, multistep process that requires commitment, and it is critical to have motivated individuals. Our study had a 100% response rate over the three rounds, and we are grateful to those who gave up their time to participate in our work.

The WSI-based statements were generally well received, however, some of the participants highlighted that the purpose of these images was not adequately communicated. Some participants were under the impression that they were being assessed and this may have unduly influenced their response(s). Furthermore, while WSIs were considered superior and more in-keeping with routine practice compared with photomicrographs, the lack of access to additional levels and immunohistochemistry was a limitation. In addition, some participants reported problems with the resolution of the images. The potential role of digital pathology and artificial intelligence in improving accuracy of the assessment of the degree of luminal occlusion was briefly explored. Current evidence demonstrates that artificial intelligence can improve the high interobserver variability commonly seen in biomarker evaluation (ie, estimation of Ki67 scoring in breast cancer and tumour-infiltrating lymphocyte scoring in melanoma).^29^ The participants agreed that the pathologist’s visual assessment is subjective in assessing the degree of luminal occlusion, and they feel that there is scope to reduce the interobserver variability of this assessment through the increasing use of digital pathology.

## Conclusions

The Delphi method in histopathology is a valid and reliable research method, which can be applied to traditionally understudied areas, such as GCA. This is the first time that a Delphi study has been undertaken to resolve areas of disconcordance in pathology practice for GCA and we have demonstrated the potential benefits and acceptability of a standardised reporting protocol to ensure consistent reporting practices and facilitate data collection.

By bringing together geographically dispersed expert pathologists in an online, multistage process, our study has shown that there are key areas of consensus in this challenging field that can be used to develop a standardised reporting protocol for TAB specimens. Finally, this Delphi method has highlighted where evidence is limited and/or consensus cannot be reached, and thus we propose the need for future research to clarify these key areas in GCA diagnosis and management.

## Data Availability

Data are available on reasonable request.
