# Supplementary material for: Histopathology reporting of temporal artery biopsy specimens for giant cell arteritis: results of a modified Delphi study
Source: J Clin Pathol. 2023 Jun 15;77(7):464–70. doi: 10.1136/jcp-2023-208810 (PMC11228225; doi:10.1136/jcp-2023-208810)
Supplement: Supplementary data [file jcp-2023-208810supp001.pdf]

Supplementary information for the formulation and discussion of Delphi statements

Methodology

Whole slide image selection

The WSIs were selected by two of the study co-ordinators (DT and AC). In addition, 5 photomicrographs were selected to represent various degrees of luminal occlusion. Clinical information, immunohistochemistry, further histological levels, and special stains such as Elastic Van Gieson (EVG) were not provided. Statements were formulated based on the key histological features seen in the images and the participants were asked to view the slides via the Leeds Virtual Pathology website (LVP)[17]. All participants were advised that the purpose of this exercise was to stimulate discussion and highlight areas of disconcordance, rather than to assess the participant's knowledge of specific pattern types or other diagnostic parameters.

Leeds online surveys

Ethical approval for the study was obtained from the research ethics committee of the Faculty of Medicine and Health, University of Leeds (MREC 21-012) and each panel member provided written consent for their participation in the Delphi process. Unique login credentials were provided to each participant for the purpose of maintaining anonymity. Only the study co-ordinator was unblinded to the individual responses for administrative and feedback purposes.

Results

Here we provide summaries of the key discussion points from the expert participants.

Clinical information

In routine clinical practice, it can be difficult for the pathologist to obtain all the relevant background information before assessing a TAB. Consensus was reached regarding the

following information being provided by the clinician on the request form: degree of clinical suspicion for GCA, presenting signs and symptoms, relevant past medical history (i.e., polymyalgia rheumatica), laboratory markers of inflammation, and the dose and start date of corticosteroid therapy. The participants felt that knowledge of these parameters may help with nuanced histological interpretation, although it was debated to what degree they correlate with or predict histological features.

One clinical parameter which did not reach consensus was the need to include the temporal artery ultrasound (US) scan result on the request form (5). The collective experience of the panel was that if the clinician provides the US findings, they may use them for the purposes of clinicopathological correlation, but they would not routinely seek out this information (using electronic health records for example). Whilst there is recent literature supporting the use of US imaging as the first-line test for patients with suspected GCA[3], some of the participants were not aware of their centre adopting this practice or if routine US was even accessible to their clinicians.

Specimen handling and tissue pathways

Most of the statements in this section were formulated based upon current recommendations in the RCPath tissue pathway for cardiovascular pathology[11]. Consensus was achieved on many statements in this category, however; there were consistent areas of debate in all 3 survey rounds which could not be resolved, and these statements (10 ,17) were subsequently omitted.

The participants agreed on the general best practice guidelines for TAB specimen handling and processing in the laboratory (i.e., the minimum length of biopsy post-fixation, the plane of sectioning, and embedding the sections “on end” rather than transversely). There was

unanimous agreement for submitting the entirety of the biopsy for histology given the patchy nature of GCA (14).

Consensus could not be reached for the RCPath recommendation to comment on the presence of tortuosity at macroscopy (10). The participants felt that this information is uninformative for histological interpretation and speculated whether it is a term more usually used by clinicians as a surrogate marker for suspicion for GCA.

A particularly contentious topic concerned the suggestion of completely exhausting a tissue block before definitively calling a biopsy negative for GCA (17). The participants could not justify the routine adoption of this practice due to time pressures and finite resources, and the panel questioned its diagnostic value. There was, however, support for obtaining additional levels until the pathologist is satisfied that the tissue has been "adequately examined" (81). Consensus on the number of levels was not specified, however – in drawing from their own experience – the expert panel felt that an additional 12 levels would generally be deemed sufficient.

Initial histological investigations

All participants agreed that three histological levels should be ordered "up-front" at the time of cut-up (16) and that initial H&E-stained sections only are sufficient for morphological assessment (15). Whilst most participants agreed that an EVG stain can be a useful adjunct in assessing for breaks, loss, or duplication in the internal elastic lamina (25), it was not deemed feasible or appropriate to order an up-front EVG on all cases (18). Instead, participants agreed that the use of additional stains should be dictated by the pathologist after review of the initial H&E sections (19) and furthermore that immunohistochemistry (specifically CD68 and CD3 staining), is not routinely required (27,28).

Periarteriolar lymphocytic infiltration - PALI

The participants agreed that isolated aggregates of chronic inflammatory cells around the small vessels surrounding the main vessel (periarteriolar lymphocytic infiltration – PALI), in the absence of inflammation in the main artery wall, is not a diagnostic feature of GCA (40). In such instances, the pathologist should be advised to undertake further laboratory investigations i.e., serial levels and/or exhaustion of the tissue block (42). Furthermore, the panellists supported the notion that the significance of PALI is uncertain but that, in the correct clinical setting, may prompt consideration of alternative diagnoses (41). The group preferred to not define the potential differential diagnoses for PALI given the limited evidence base, and instead advocate for clinicopathological correlation. The participants did not support a mandated discussion of these cases at a clinicopathological meeting or equivalent (82); instead, advocating that pathologists communicate their diagnostic uncertainties in their own preferred manner.

Multidisciplinary team meetings

Some of the participants highlighted that whilst it may be best practice to discuss challenging cases at a multidisciplinary team (MDT) meeting (47), these are not routine in the NHS. The panel felt that establishing new GCA MDT meetings is not currently feasible given the many various external pressures on staffing and pathology workloads. The group agreed however, that if the need arises, they would prefer direct discussion between the pathologist and referring clinician.

Nomenclature

The panel agreed that chronic inflammation (macrophages and/or lymphocytes) is a consistent histological finding in GCA (21), although there can be considerable variation in the extent of inflammation seen in TAB sections (29). Several participants admitted that the terminology used for their final diagnosis is dependent on the presence or absence of giant

cells. For example, some participants use the term 'giant cell arteritis' if giant cells are observed on TAB, and 'temporal arteritis' if giant cells are absent. The panellists acknowledged that whilst these terms are often used interchangeably, they felt that standardised terminology is important to reduce inconsistencies and proposed the term 'temporal (giant cell) arteritis'.

Establishing reproducible definitions for 'healing/healed/resolving/treated' arteritis

The participants felt that the terms 'healing/healed/resolving/treated' arteritis lack clear and agreed definitions (44), and they prefer instead to describe the histological features seen without drawing definitive conclusions in their reports (83). Several participants stated that these terms are overused in reporting and felt that consensus should be obtained as to (a) whether these are in fact recognisable entities, and (b) if so, what criteria should be used to arrive at these diagnoses.

The reporting of oedema and neoangiogenesis

The reporting of oedema was not supported by participants as many find it difficult to assess and ultimately it was felt that this feature has no known diagnostic or clinical utility (33). Some of the participants did not support the routine reporting of neoangiogenesis (36) as they felt that the implications of its presence/absence were not supported by firm evidence. Others expressed the view that there could be potentially unknown associations between neoangiogenesis and clinical outcomes, and it might be useful to capture this information as a non-core item in a reporting proforma.

The effect of glucocorticoid therapy on TAB interpretation

The statement (46) pertaining to whether, in the short term (<1 month), corticosteroid therapy at full doses influences the histological appearances at TAB, sparked considerable debate and discussion amongst the panel. Most participants stated that, in their experience, there is no

consistent observation made between duration of steroid therapy treatment and the degree of chronic inflammation seen. Some panellists felt that steroid therapy initiates rapid changes (within days) in the biopsy findings whereas others thought that the inflammatory changes can persist for weeks despite steroid treatment. Overall, the participants agreed that more research is needed in this area.

Atherosclerosis and/or age-related changes

A WSI statement which stimulated important discussion, concerned the possible consideration of atherosclerosis related changes as a differential diagnosis (75) in TAB specimens for GCA. The group agreed that there are no clear, established, histological characteristics for age-related changes and atherosclerosis, making it challenging to distinguish these entities on microscopy (84). There was also agreement that breaks, fragmentation, and/or loss of the internal elastic lamina are not specific features for GCA (26) and may be seen as age-related phenomena. Some participants stated that they would prefer to order an EVG stain to assess the extent of disruption in the elastin layer in such cases, but this approach was not shared by the majority. Whilst some participants were happy to regard these two entities as part of a spectrum of changes and did not view distinction crucial, others were in favour of adding this as a topic for further research.

Whole slide image-based statements

Consensus was reached on the photomicrographs that illustrated the various degree of luminal occlusion (76-80). Despite this, the participants were in favour of reporting the degree of luminal occlusion by broadly specifying if it is <50%, >50%, or completely occluded (35). The participants expressed uncertainty of the clinical benefit of attempting to specify the degree of luminal occlusion by smaller margins, and referenced (as an example),

the already established poor inter-observer variability of this exercise in the macroscopic assessment of coronary artery stenosis at post-mortem.

Whilst consensus was reached that the participants are aware of the four validated histological patterns of GCA (38), several individuals stated that they do not have enough practice and familiarity with these categories to routinely "commit" to a pattern. The exception to this was the WSI example of "panarteritis pattern GCA" which achieved 100% consensus (52). Discussions were held regarding the mechanism of GCA progression proposed by Hernández-Rodríguez *et al*[12]. The authors propose that the different patterns of GCA represent a continuum of disease progression rather than distinct categories. Given that there may therefore be overlap between these patterns, some participants questioned the value of attempting to formally categorise the pattern on a TAB. Other members of the panel described how, in their personal experience, the panarteritic pattern seems to correlate closely with the ultrasound findings. In conclusion, the participants agreed that research is needed to explore if, and how, the histological pattern types correlate with various clinical parameters and ultimately with patient outcome.

Supplementary Figures

Figure 1s: Results from the Royal College of Pathologists consultant body survey with A) depicting the number of cases reported by the survey participants and highlighting that 61% of histopathologists report only 0-10 temporal artery biopsy specimens annually. Panel B) summarises the expressed opinions relating to the histopathological criteria for GCA reporting and perceived lack of current guidelines.

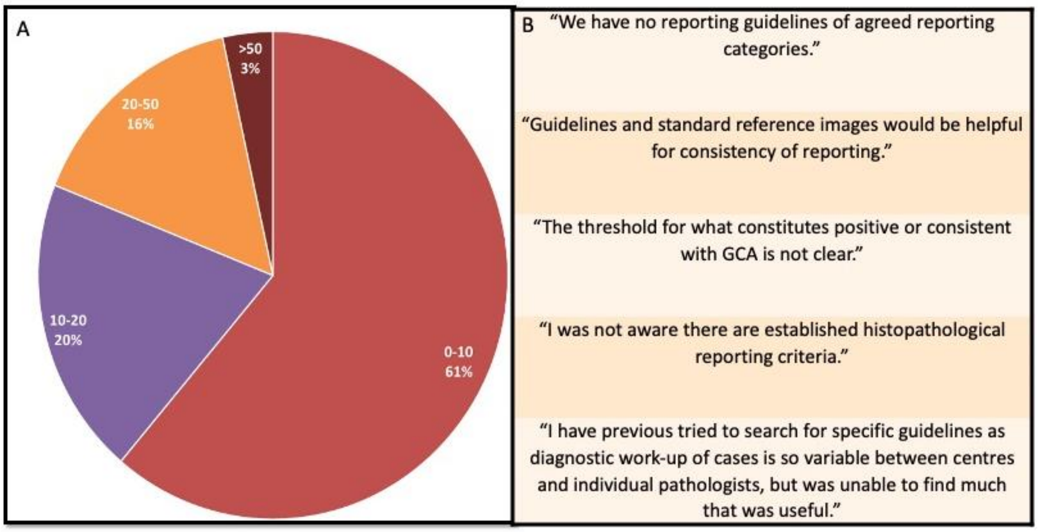

Figure 2s: Illustration of the Likert scale used

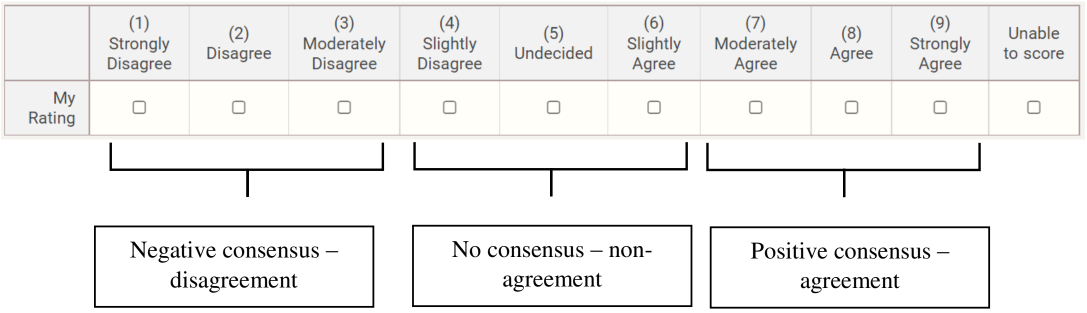

Consensus was defined a priori and considered to have been reached if ≥70% of the expert participants selected moderately agree/agree/strongly agree (agreement - positive consensus; Likert ratings 7-9) or moderately disagree/disagree/strongly disagree (negative consensus – disagreement; Likert ratings 1-3). Ratings 4-6 were considered non-agreement.

Figure 3s: Example of feedback provided to the participants for the statements which did not reach consensus. The group response distribution represents the proportion (%) of agreement (green), no agreement (yellow) and disagreement (red). Each participant's own rating for each statement was also provided alongside any anonymised free-text responses.

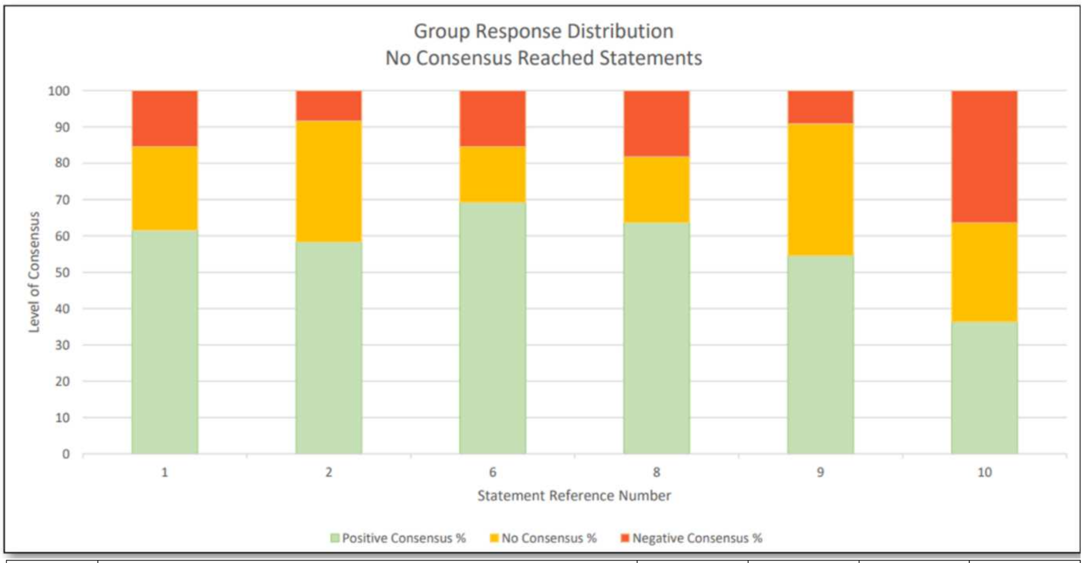

Statement No.	Statement	Individual Agreement Score	Group Agreement - Positive Consensus (%)	Group Agreement - No Consensus (%)	Group Agreement - Negative Consensus (%)
1	If the patient has undergone ultrasound of the temporal artery/ies then a summary of the radiological findings can be helpful if provided on the request form	8	62	23	15

Supplementary Table

Table 1s: Complete list of statements that reached consensus. Additional statements added by the expert participants are provided at the end of the table.

No.	Statement	Agreement
**Category 1: clinical information**		
1	The clinician should clearly state their degree of suspicion for GCA on the request form	77%
2	Details of the patient's presenting clinical signs and symptoms should be mentioned on the request form	92%
3	Important relevant past medical history, for example hypertension or polymyalgia rheumatica, should be mentioned on the request form	92%
4	Laboratory markers of inflammation, specifically CRP and/or ESR, can be helpful if stated on the request form	92%
6	If the patient is on corticosteroid therapy, then the dose and start date should be given on the request form	77%
**Category 2: specimen handling and tissue pathways**		
9	The length of the biopsy, post fixation, should be at least 5-10mm	83%
11	The biopsy should be sectioned transversely into segments 3-5mm long	73%
12	It is appropriate for up to a maximum of three pieces of tissue to be placed into the same cassette	70%
13	All the transverse sections should be embedded "on end"	100%
14	The entirety of the biopsy should be submitted for histology as GCA may be focal or patchy	100%
15	Initial assessment by H&E stain only is sufficient	73%
16	Three histological levels should be requested upfront at the time of cut-up	100%
18	Ordering an EVG stain, after examining the initial H&E sections, might be helpful	91%
19	The use of additional stains depends on what is visible on the H&E	73%
**Category 3: microscopic assessment and reporting**		
20	Scanning at low power (2x or 4x magnification) should be used initially to assess for the presence of inflammation	73%
21	Chronic inflammation (macrophages and/or lymphocytes) is a consistent histological finding in GCA	85%
22	The presence of multinucleated giant cells is helpful, but not required, for the diagnosis of GCA	92%
23	It is important to specify the extent of any granulomatous infiltration in the artery wall	75%
24	It can be useful to comment on whether the tunica media is intact or disrupted	85%
25	An EVG stain can be helpful to assess for breaks, loss, or duplication in the internal elastic lamina	91%
26	Breaks, fragmentation, or loss of the internal elastic lamina are not specific features for GCA	83%
27	Immunohistochemistry for histiocytic markers, such as CD68, is not routinely required	83%
28	Immunohistochemistry for lymphocytic markers, such as CD3, is not routinely required	91%
29	When examining temporal artery biopsies, there is considerable variation in the extent of inflammation	100%
30	The location of inflammatory cells in the artery wall should be included in the report	100%
31	The presence or absence of intimal hyperplasia should be reported	83%
32	The presence or absence of fibrosis should be reported	83%
34	The presence or absence of luminal occlusion should be reported	92%
35	It is useful to comment on the degree of luminal occlusion by broadly specifying if it is <50%, >50% or completely occluded	92%
38	I am aware that there are 4 validated histological patterns of GCA	85%
39	Using a classification system which recognizes the different patterns of GCA is	75%

	important for improving standardization of the reporting of GCA	
40	Isolated aggregates of chronic inflammatory cells around the small vessels surrounding the main vessel, in the absence of inflammation in the main artery wall, is not a diagnostic feature of GCA	77%
41	The significance of PALI is uncertain but in the correct clinical setting, alternative diagnoses may warrant consideration	83%
42	Isolated aggregates of chronic inflammatory cells around the small vessels surrounding the main vessel, without inflammation involving the main artery wall, should prompt further laboratory investigation i.e., levels and/or exhaustion of the tissue block	85%
43	It can be difficult to differentiate GCA from other systemic vasculitides	83%
44	I find the terms 'healing/healed/healed/treated' arteritis lacking in clear definitions	77%
**Category 4: whole slide image-based statements**		
48	Scanning at low power magnification, there is lymphocytic infiltration in all layers of the artery wall	100%
49	Giant cells, occurring occasionally in aggregates, are readily identifiable at the intima/media junction	100%
50	There is evidence of periarteriolar lymphocytic inflammation of the vasa vasorum and distant blood vessels	80%
51	There is intimal hyperplasia	100%
52	I would report this is panarteritic pattern GCA	100%
53	There is no evidence of inflammation in these sections	70%
54	These temporal artery biopsies are within normal limits	70%
55	On initial overview, there is lymphocytic infiltration in all layers of the artery wall	100%
56	There is focal destruction of the tunica media	70%
57	Neoangiogenesis is observed in the intima	90%
58	There is evidence of periarteriolar lymphocytic inflammation of the vasa vasorum and distant blood vessels	70%
59	I would classify these sections as consistent with temporal (giant) cell arteritis even though I cannot see giant cells and/or granulomatous infiltrate	73%
61	There is evidence of periarteriolar lymphocytic inflammation of the vasa vasorum and distant blood vessels	100%
62	The tunica media is partially disrupted	80%
64	Lymphocytic infiltration is seen in the adventitia with local infiltration into the media	100%
65	There is focal destruction of the media	90%
67	Lymphocytic infiltration is seen in the adventitia and intima only, without involvement of the media	90%
68	Fibrosis is observed in these sections	80%
69	There is intimal hyperplasia	100%
70	I would report this as concentric bilayer pattern GCA	70%
72	Fibrosis is observed in some of these sections	90%
74	The internal elastin layer is discontinuous in places	80%
76	There is no evidence of luminal occlusion	90%
77	The grade of luminal occlusion is <50%	90%
78	The grade of luminal occlusion is 50-75%	90%
79	The grade of luminal occlusion is 75-100%	100%
80	There is complete luminal occlusion	90%
**Statement that reached negative consensus (disagreement)**		
60	Lymphocytic infiltration is seen in the adventitia only	70% disagreement
**Additional statements added by the expert participants**		

81	If the first three levels are inconclusive, additional levels (three or more) should be requested until the pathologist is satisfied that the tissue has been adequately examined	91%
83	Instead of using the terms 'healing/resolving/healed/treated' arteritis, I prefer to describe the histological features seen in my report without drawing definitive conclusions	83%
84	There is overlap in the histological features for age-related changes and atherosclerosis, making it difficult to distinguish between these entities	73%
